# Relationship Between Lifestyle, Health Factors, and Severity of Lower Urinary Tract Symptoms Amongst Men Living in Southeast Nigeria

**DOI:** 10.7759/cureus.72188

**Published:** 2024-10-23

**Authors:** Chiedozie Chineme-Anyaeze, Ezinne E Ijiomah, Chineme M Anyaeze, Osaze Ehioghae

**Affiliations:** 1 Urology, Six-C Specialist Clinic, Owerri, NGA; 2 Urology, Federal University Teaching Hospital Owerri, Owerri, NGA

**Keywords:** benign prostatic hyperplasia (bph), international prostate symptom score (ipss), lower urinary tract symptoms (luts), male luts, nigeria, owerri, urology

## Abstract

Background

Lower urinary tract symptoms (LUTS) refer to a group of symptoms related to the storage and voiding of urine that comprises storage, voiding, and post-micturition symptoms affecting the lower urinary tract, and the prevalence in men in our society is on the rise as a result of an increase in the life expectancy. This study investigates the relationship between lifestyle, health factors, and severity of LUTS amongst men living in the southeastern part of Nigeria.

Method

A cross-sectional study of 389 men aged 42-96 years was conducted. The severity of LUTS was assessed using the International Prostate Symptom Score (IPSS). Health factors such as diabetes mellitus, hypertension, age, prostate volume, prostate-specific antigen, body mass index, and others were examined using the chi-square test.

Results

Out of the 389 participants enrolled in the study, the mean age was 69.21 ± 9.683 years. Nocturia was the most reported symptom in 337 participants. Over half of the respondents (212) experienced severe symptoms, while 152 (39.1%) reported moderate issues, and 25 (6.4%) had mild symptoms. Mean values were as follows: prostate volume of 69.62 ± 34.82 g, post-void residual urine of 52.72 ± 14.96 mL, and prostate-specific antigen (PSA) of 23.42 ± 28.78 ng/mL. Using the chi-square test, significant associations were found between LUTS severity and age (x^2^=37.454; p≤0.001), diet (x^2^=16.341; p=0.003), BMI (x^2^=21.152; p=0.002), diabetes mellitus (x^2^=7.690; p=0.021), prostate volume (x^2^=22.001; p≤0.001), and post-void residual urine (x^2^=10.779; p=0.005).

Conclusion

This study highlights the importance of socio-demographic characteristics, lifestyle factors, and clinical parameters in predicting LUTS severity. These findings can inform prevention and management strategies for LUTS.

## Introduction

Lower urinary tract symptoms (LUTS) refer to a group of symptoms related to the storage and voiding of urine that comprises storage, voiding, and post-micturition symptoms affecting the lower urinary tract. There are many possible causes of LUTS, such as abnormalities or abnormal function of the prostate, urethra, bladder, or sphincters. In men, the most common cause is benign prostate enlargement (BPE), which obstructs the bladder outlet. Other conditions that can cause LUTS include detrusor muscle weakness or overactivity, prostate inflammation (prostatitis), urinary tract infection, prostate cancer, and neurological disease [1].

LUTS are a major burden for the aging male population. Age is an important risk factor for LUTS, and the prevalence of LUTS increases as men get older. Bothersome LUTS can occur in up to 30% of men older than 65 years. This is a large group potentially requiring treatment[1].

The number of older people is growing at the fastest rate in Africa, followed by Latin America, the Caribbean, and Asia; nearly 80% of the world’s older population will reside in less developed countries by 2050. Thus, the aging issue is inevitable for all countries[2].

The prevalence of male LUTS is becoming high in a typical Nigerian southeastern society such as Owerri due to the increasing aging population, between 1998 and 2019, life expectancy and HALE (Health Adjusted Life Expectancy) increased in Nigeria by 18% to 64.3 years [3]. The investigator therefore proposes that, with the increasing elderly population, there should be a corresponding increase in the prevalence of male LUTS, which has justified the need for this study in our environment.

Lifestyle and health factors, such as advancing age, family history, obesity, diabetes mellitus, hypertension, smoking body mass index, and others, have been shown to have an impact on the severity of male lower urinary tract symptoms [4-7]. However, not many studies of this kind have been done in our locality. In Nigeria, the prevalence of these risk factors is compounded by limited access to healthcare, cultural barriers, and socioeconomic disparities. The interplay between these factors and LUTS severity is poorly understood, particularly in southeastern Nigeria.

This study aims to investigate the pattern of male LUTS in Owerri, Nigeria, with a focus on identifying key health factors and their associations with LUTS severity. We postulate that modifiable risk factors, such as obesity and smoking, contribute significantly to the LUTS burden in this population. Our findings will inform evidence-based interventions, healthcare policy, and clinical guidelines tailored to the unique needs of Nigerian men.

## Materials and methods

Study population

This research was carried out in the urology unit of Federal Teaching Hospital Owerri (public hospital) and Six-C Specialist Clinic (a private urology center), Owerri, Imo state, Nigeria, between November 2023 and April 2024. Imo state is situated in Southeastern Nigeria with Owerri as its capital. Owerri has three local government areas, namely, Owerri Municipal, Owerri West, and Owerri North. The public hospital, a tertiary health facility in Owerri, is situated in Owerri Municipal, while the private hospital is in Owerri West. The population of Owerri is about 400,000[8].

Study design

This is a hospital-based prospective descriptive cross-sectional study.

Sample selection

Men aged 40 years and above who presented to the urology clinic with LUTS and consented to participate in the study were recruited. Men aged 40 years and above who attended urology clinics during the study period, provided informed consent to participate, and presented with LUTS were included in the study, while men below 40 years of age, women with LUTS, and men above 40 years who did not provide consent were excluded from the study.

Sample size calculation

The minimum sample size was estimated using the formula for prevalence studies [9].

n = $\frac{z^{2}pq}{d^{2}}$

Where n = minimum sample size

Z = standard normal deviation taken to be 1.96 for studies of this nature, which corresponds to a 95% confidence level.

P = Estimated proportion of men with LUTS in the community in a recent community-based cross-sectional study (Kim et al. determined a prevalence rate of 36% for LUTS in men aged 40 years and above in Imo state[10]).

Thus, P=0.36

q = (1-P)

q = 0.64

d = level of precision of 0.05

Thus, the minimum sample size is 354.

A further 10% (35) was added to the minimum sample size calculated to ensure that the study is adequately powered even when attrition occurs through withdrawal from the study or refusal of consent.

Therefore, 389 men were recruited for the study.

Ethical consideration

Ethical approval was obtained from the Health Research Ethical Committee of Federal Teaching Hospital, Owerri, and informed consent was obtained from participants. Confidentiality and anonymity were maintained.

Study procedure

A detailed history and full physical examination were conducted on all recruited subjects. The International Prostate Symptom Score (IPSS) was utilized to assess the severity of LUTS. Medical records were reviewed to obtain past medical history. Blood tests and imaging studies were performed, and data were collected using the study's designed questionnaire.

Data analysis

Data were coded and analyzed using Statistical Product and Service Solutions (SPSS, version 21.0; IBM SPSS Statistics for Windows, Armonk, NY). Frequency tables, charts, and figures were used to summarize as required. Mean, median, and standard deviation were calculated for quantitative variables, while proportions were generated for categorical variables. Chi-square and ANOVA tests were used to test for statistical associations among categorical variables where appropriate. A 95% confidence interval and p-value of <0.05 were considered statistically significant.

## Results

Sociodemographic

Table 1 presents the sociodemographic characteristics of the 389 participants enrolled in the study. The majority of the participants (273, 70.2%) were aged 61-80 years, followed by 40-61 (19.5%). The mean age was 69.21 ± 9.683 years. Most of the participants 371 (95.4%) were married, three (0.8%) were divorced, and two (0.5%) were single. The majority of the participants (198, 50.9%) were retired, followed by business owners (63, 16.2%), and civil servants (40, 10.3%).

**Table 1:** Sociodemographic characteristics of the study participants.

Variables	Categories	N=389 (%)
Age	40-61	76 (19.5)
	61-80	273 (70.2)
	>80	40 (10.3)
Marital Status	Single	2 (0.5)
	Married	371 (95.4)
	Divorce	3 (0.8)
	Widower	13 (3.3)
Occupation	Civil Servant	40 (10.3)
	Business	63 (16.2)
	Farmer	31 (8.0)
	Self-employed	36 (9.3)
	Artisan	3 (0.8)
	Retired	198 (50.9)
	Unemployed	18 (4.6)
Nationality	Nigerian	389 (100)
	Non-Nigerian	0 (0.0)
State	Abia	10 (2.6)
	Akwa-Ibom	3 (0.8)
	Anambra	2 (0.5)
	Ekiti	2 (0.5)
	Imo	370 (95.1)
	Ondo	2 (0.5)

Presenting symptoms

Table 2 shows the presenting complaints. Nocturia was reported by 337 (86.6%) of the participants, a significant proportion of 324 (83.3%) of participants experienced urinary frequency, and the least reported symptom is hesitancy (152, 39.1%). The storage symptoms were more reported by the participants than the voiding symptoms.

**Table 2:** Frequency of presenting symptoms among study participants.

Symptoms	Yes, N=389 (%)	No, N=389 (%)
Frequency	324 (83.3)	65 (16.7)
Nocturia	337 (86.6)	52 (13.4)
Urgency	264 (67.9)	125 (32.1)
Feeling of Incomplete Emptying of the Bladder	268 (61.2)	151 (38.8)
Weak Urinary Stream	245 (63)	144 (37)
Hesitancy	152 (39.1)	237 (60.9)
Straining	216 (55.5)	173 (44.5)
Urge Incontinence	189 (48.6)	200 (51.4)

IPSS and quality of life

**Figure 1:**
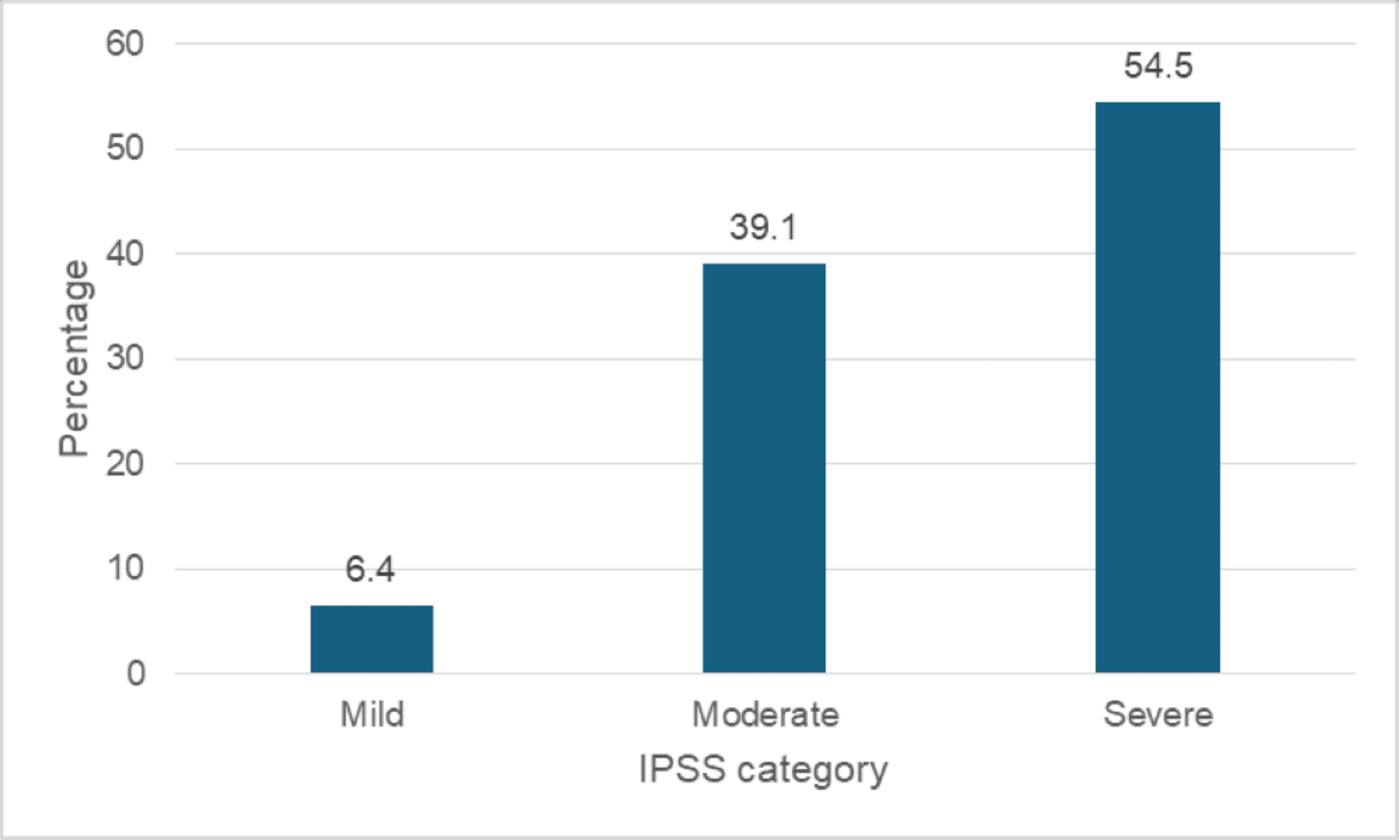
Distribution of International Prostate Symptom Score (IPSS) among participants.

**Figure 2:**
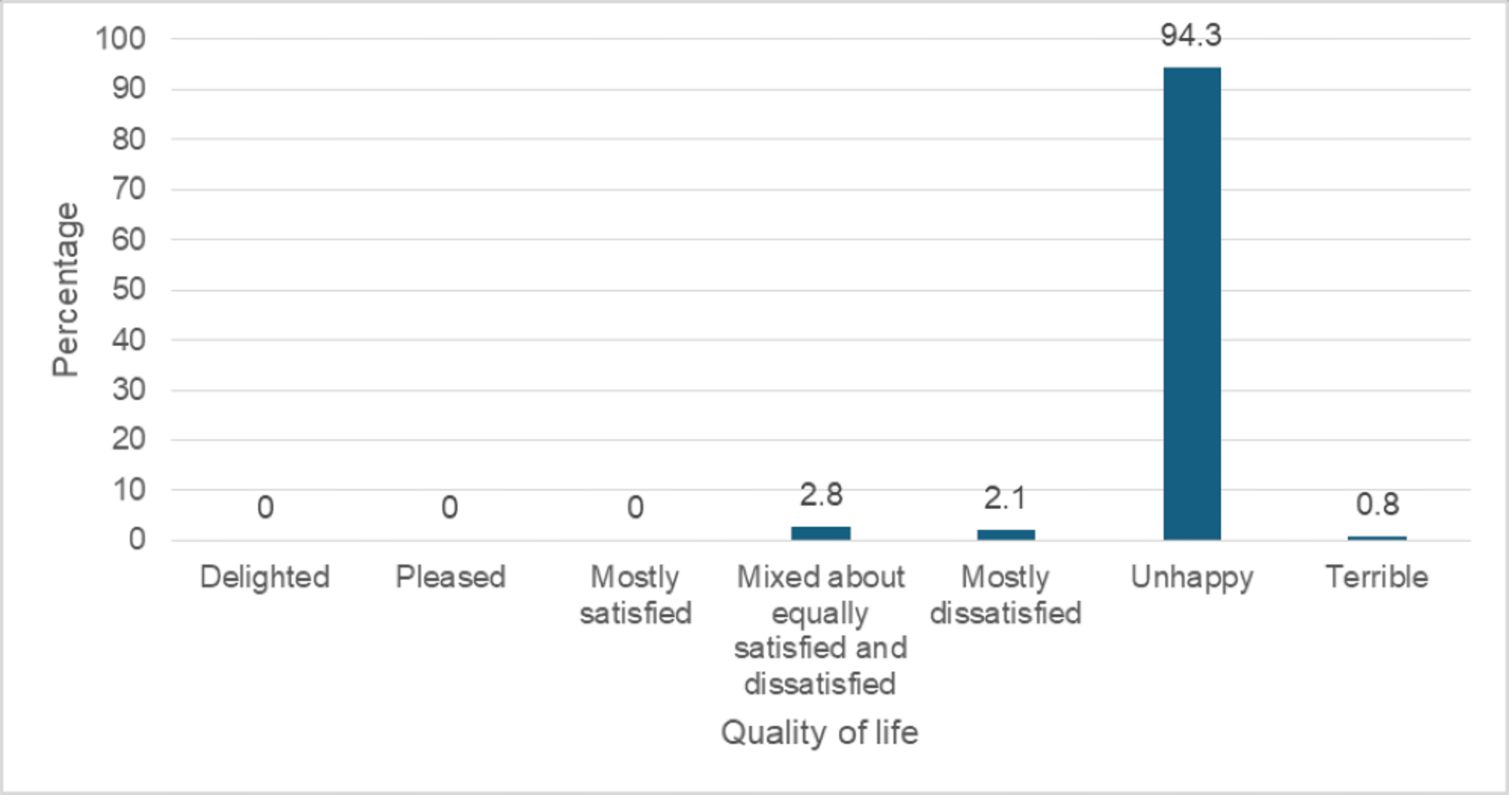
Bar chart showing the quality of life of the participants.

Figure 1 reveals that over half of the respondents (212, 54.5%) experienced severe symptoms, while 152 (39.1%) reported moderate issues, and 25 (6.4%) had mild symptoms. Regarding the quality of life in Figure 2, an overwhelming majority (367, 94.3%) reported being unhappy, with three (0.8%) describing their experience as terrible. Notably, none of the respondents expressed positive emotions, such as being delighted, pleased, or mostly satisfied, highlighting the impact of urinary symptoms on daily life. Only a small fraction (2.8%) reported mixed feelings, and 2.1% were mostly dissatisfied.

Laboratory and examination findings

The clinical and laboratory findings of the 389 participants revealed a high prevalence of obesity (180, 46.3%), prostate enlargement (383, 98.5%), and elevated prostate-specific antigen (PSA) levels (233, 59.9% ± 10 ng/mL). The majority had normal fasting blood sugar (269, 69.2%). Prostate volume was predominantly moderate (220, 56.6%: 41-100 g), while post-void residual urine was elevated (213, 54.8% ± 50 mL). Mean values were prostate volume (69.62 ± 34.82 g), post-void residual urine (52.72 ± 14.96 mL), and PSA (23.42 ± 28.78 ng/mL). Only 22 (5.7%) were underweight, and 18 (4.6%) had diabetes (Table 3).

**Table 3:** Key laboratory and examination findings of the study participants.

Variables	Categories	N=389 (%)
BMI	Underweight	22 (5.7)
	Normal	90 (23.1)
	Overweight	97 (24.9)
	Obese	180 (46.3)
DRE	Not enlarged	6 (1.5)
	Enlarged	383 (98.5)
FBS (mg/dL)	Hypoglycemia (≤69)	19 (4.9)
	Normal (70-100)	269 (69.2)
	Prediabetes (101-125)	83 (21.3)
	Diabetes (≥126)	18 (4.6)
Prostate volume (g)	≤40	106 (27.2)
	41-100	220 (56.6)
	≥101	63 (16.2)
Post void urine	<50	176 (45.2)
	≥50	213 (54.8)
PSA (ng/mL)	≤4	55 (14.1)
	4-10	101 (26.0)
	≥10	233 (59.9)
Mean prostate volume	69.62±34.82	
Mean PVR	52.72±14.96	
Mean PSA	23.42±28.78	

Relationship between sociodemographic, health factors, and severity of LUTS

This study examined the relationship between sociodemographic characteristics, risk factors, and severity of LUTS among 389 respondents. Significant associations were found between LUTS severity and age (p<0.001), diet (p=0.003), BMI (p=0.002), diabetes mellitus (p=0.021), prostate volume (p<0.001), and post-void residual urine (p=0.005). Specifically, older age, cassava-based diet, obesity, diabetes, larger prostate volume, and higher post-void residual urine were associated with increased LUTS severity. No significant associations were found for alcohol consumption, smoking, hypertension, cardiac disease, family history of BPH, or prostate cancer (Table 4).

**Table 4:** Relationship between sociodemographic, risk factors, and severity of LUTS. LUTS, Lower urinary tract symptoms

Variables	Categories				p-value	X^2^
		MILD	MODERATE	SEVERE		
Age	≤40	0 (0.0)	0 (0.0)	0 (0.0)		
	41-60	3 (12.0)	53 (34.9)	20 (9.4)	<0.001	37.454
	61-80	19 (76.0)	87 (57.2)	167 (78.8)		
	>80	3 (12.0)	12 (7.9)	25 (11.8)		
Diet	Cassava	4 (16.0)	87 (57.2)	110 (51.9)	0.003	16.341
	Grain	12 (48.0)	39 (25.7)	69 (32.5)		
	Heavy Cassava (night)	9 (36.0)	26 (17.1)	33 (15.6)		
Alcohol	Yes	5 (20.0)	38 (25.0)	43 (20.3)	0.545	1.213
	No	20 (80.0)	114 (75.0)	169 (79.7)		
Smoking	Yes	0 (0.0)	2 (2.0)	3 (1.4)	0.741	0.600
	No	25 (100.0)	149 (98.0)	209 (98.6)		
Hypertension	Yes	12 (48.0)	68 (44.7)	85 (40.1)	0.571	1.122
	No	13 (52.0)	84 (55.3)	127 (59.9)		
BMI	Underweight	0 (0.0)	14 (9.2)	8 (3.8)		
	Normal	8 (32.0)	20 (13.2)	62 (29.2)	0.002	21.152
	Overweight	3 (12.0)	40 (26.3)	54 (25.5)		
	Obese	14 (56.0)	78 (51.3)	88 (41.5)		
Diabetes	Yes	6 (24.0)	13 (8.6)	37 (17.5)	0.021	7.690
	No	19 (76.0)	139 (91.4)	175 (82.5)		
Cardiac Disease	Yes	0 (0.0)	5 (3.3)	5 (2.4)	0.603	1.011
	No	25 (100.0)	147 (96.7)	207 (97.6)		

## Discussion

This study investigated the pattern of male LUTS among 389 men aged 42-96 years in Owerri. The findings provide valuable insights into the symptoms, risk factors, and characteristics of LUTS in this population.

Our study investigated the potential association between age, diet, BMI, diabetes mellitus, and the severity of LUTS. Our study revealed a positive correlation between age and LUTS severity, as noted in several previous similar studies[5,11]. These associations may be attributed to the age-related increase in the incidence of prostatic bladder outlet obstruction due to benign and malignant prostate enlargement[12-14], isolated age-induced changes in bladder function[5], increased prevalence of comorbidities, and hormonal changes. These findings highlight the importance of age-sensitive management strategies for LUTS. There is a relative paucity of literature evaluating the association between diet and LUTS, and the findings of these studies are heterogeneous and conflicting. A 2008 study by Kristal et al. that analyzed the incident cases of BPH among patients assigned to the placebo arm of the prostate cancer prevention trial found that a diet that included moderate alcohol intake and was high in protein and vegetables and low in fat and red meat was protective for LUTS[15]. A study by Chyou et al. found that beef intake slightly increased the risk of LUTS, although an analysis of 32 other food groups found no increased risk [16]. Analysis of the health professional's follow-up study found that vegetable intake was inversely correlated with LUTS, but other food groups showed no strong association [17]. In other studies, starch, vegetable consumption, fat consumption, and poultry have been shown to increase the risk of BPH and LUTS[18], whereas others have shown vegetables and unsaturated fats to be protective from LUTS [19]. This study shows that cassava-based and grain-based diets are associated with increased LUTS severity, which are commonly consumed food products in the southeastern part of Nigeria. In the Nigerian context, dietary patterns are influenced by cultural and socioeconomic factors [20]. The heterogeneous patient populations, definitions used for LUTS, and analytic methods used make it difficult to draw any clear conclusions[19]. Our findings emphasize the need for culturally tailored dietary interventions. The association between BMI and LUTS severity observed in our study aligns with previous research by Penson et al. [7], which demonstrated a significant link between obesity and moderate-to-severe LUTS. Similarly, our study found a significant relationship between BMI and LUTS severity (p-value=0.002), with 180 (46.3%) of participants being obese. However, our findings contrast with the study by Nnabugwu et al.^. ^[21], which reported no significant association between BMI and LUTS. This discrepancy may be attributed to differences in the study population, design, and sample size; in addition, variations in BMI classification, LUTS assessment tools, or statistical analysis methods might contribute to the divergent results. Diabetes mellitus has been found to also be significantly associated with LUTS severity, consistent with previous research[7]. Possible mechanisms include increased abdominal pressure, inflammation, metabolic changes, neurogenic bladder, and vascular dysfunction. The high prevalence of diabetes mellitus in Nigeria [22] highlights the need for integrated management of diabetes mellitus and LUTS.

Interestingly, our study revealed no significant associations between LUTS severity and alcohol consumption, smoking, hypertension, and history of cardiac disease using chi-square analysis. These findings align with some studies[6,21] but contradict others[23-25]. The lack of association between alcohol consumption and LUTS severity in our study and others[25] suggests that moderate drinking may not significantly impact LUTS. However, heavy drinking has been linked to increased LUTS severity [4], indicating a potential dose-response relationship. Regarding hypertension and cardiac disease, conflicting findings may be due to differences in study populations or the presence of underlying comorbidities. Some studies suggest these conditions increase LUTS risk, while others find no independent association [21]. Our study highlights the importance of considering individual patient characteristics and potential confounding factors when evaluating LUTS risk factors. Future research should investigate the temporal relationships between these factors and LUTS development, exploring potential mechanisms underlying these associations.

The majority of the participants (337, 86.6%) experienced nocturia, followed by frequency (324, 83.3%), while hesitancy was the least reported symptom (152, 39.1%). This pattern mirrors previous studies [5] highlighting storage symptoms as the commonest symptom, followed by voiding and mixed symptoms. It is important to distinguish between these symptoms because different LUTS subtypes require distinct treatments ensuring adequate targeted therapy, and treatment efficacy also increases when addressing specific symptoms, thereby avoiding unnecessary treatments and minimizing potential side effects and cost. Nevertheless, accurate diagnosis of LUTS subtypes poses a significant difficulty, especially in a resource-poor society such as ours where there are limited subtype-specific diagnostic instruments.

LUTS significantly impacted the quality of life of the participants, 367 (94.3%) of the participants were unhappy about the symptoms when compared to other studies, which might be due to late presentation to the hospital that underscores the need for effective management strategies to mitigate the burden of LUTS on patients’ lives. This is however somewhat different from a related study revealing that 572 (72%) of the participants who responded to the quality-of-life score had low scores (<3) corresponding to a good quality of life despite their LUTS. Of the remaining participants, 184 (28%) had a high score (≥ 3), indicating that they considered their LUTS troublesome [5]. The IPSS score effectively stratified patients into mild, moderate, and severe categories. An overwhelming majority of the participants (212, 54.5%) had severe LUTS, which contradicts a similar study conducted at Ibadan where 53 (8.1%) of the participants were asymptomatic[5]; of the remainder, 432 (66%) were mildly symptomatic, 133 (20.3%) were moderately symptomatic, and 37 (5.6%) had severe symptoms [5]. Among these 267 respondents, 111 (41.6%) reported mild LUTS (IPSS 1-7), 126 (47.2%) reported moderate LUTS, and 30 (11.2%) reported severe LUTS[5]. This classification aligns with the American Urological Association AUA guidelines. Our findings support the use of IPSS as a reliable tool for assessing LUTS severity and guiding clinical decision-making.

This study has some limitations that should be considered when interpreting the findings; the study’s cross-sectional design limits our ability to establish causality and temporal relationships between variables. Hospital-based recruitment may introduce selection bias, as participants may have more severe symptoms or comorbidities. Additionally, the absence of a control group limits our ability to compare LUTS prevalence and severity with asymptomatic men. These limitations underscore the need for longitudinal studies to establish the temporal relationships and the use of control groups for comparative analysis.

## Conclusions

This study provides invaluable insights into the pattern of male LUTS in Owerri, Nigeria. Our findings highlight the significant associations between LUTS severity and age, diet, BMI, and DM while revealing no significant relationships with alcohol consumption, smoking, coffee, hypertension, and cardiac disease. These results underscore the importance of holistic management strategies, incorporating lifestyle modifications and integrated care, tailored to the unique sociocultural context of Owerri. The high prevalence of LUTS in this population necessitates increased awareness, early screening, and timely intervention to improve quality of life and reduce morbidity. Future research should focus on longitudinal studies, interventional trials, and exploration of novel risk factors to further elucidate the complexities of LUTS in Nigerian men. Ultimately, this study contributes to the growing body of evidence informing healthcare policy and clinical practice in Nigeria, ensuring that healthcare providers are equipped to address the unique needs of men suffering from LUTS in Owerri and beyond.
